# Is there a secular trend regarding puberty in children with down syndrome?

**DOI:** 10.3389/fendo.2022.1001985

**Published:** 2022-11-15

**Authors:** Furkan Erdoğan, Ayla Güven

**Affiliations:** ^1^ University of Health Sciences, Zeynep Kamil Women and Children Hospital, Pediatric Clinic, Istanbul, Turkey; ^2^ University of Health Sciences, Zeynep Kamil Women and Children Hospital, Pediatric Endocrinology Clinic, Istanbul, Turkey; ^3^ Pediatric Endocrinology, Istanbul Hospital, Baskent University Medical Faculty, Istanbul, Turkey

**Keywords:** down syndrome, obesity, hypothyroidism, puberty, precocious puberty

## Abstract

**Introduction:**

There are very few studies on the age of onset and end of puberty in children with Down syndrome (DS). Also, data regarding the course of puberty in these children compared to their healthy peers is limited. Moreover, there is limited information regarding the effects of factors such as obesity and hypothyroidism on the puberty process in children with DS. Our aim in our study is to determine whether the pubertal development of children with DS differs from their healthy peers and from previous studies conducted with DS children.

**Methods:**

The medical records of DS children were examined retrospectively. The anthropometric measurements and the age of onset of pubertal stages, and menarche were recorded. The patients’ age at puberty onset, the puberty processes, and age at menarche were compared with their healthy peers and previously published data on children with DS.

**Results:**

Of the 140 Down syndrome patients followed in our clinic, 51 of whom with puberty constituted the study group. The mean age of onset of puberty was 10.3 ± 1.0 years in our group (10.0 ± 0.8 years for girls, 10.6 ± 1.2 years for boys, respectively). Obesity occurred in 46% of pubertal girls with DS. The age of menarche in girls with DS was 11.8 ± 0.7 years. The menarche age of girls with DS was significantly different from healthy girls. In the DS boys, only the Tanner V stage ages were different from the healthy children. True- precocious-puberty was detected in three children.

**Conclusion:**

Although breast development begins later in females with DS than in their healthy peers; menarche is detected earlier than in their peers and a tendency towards obesity in the whole population. While the age of pubertal onset was similar to healthy children in male patients, our findings suggest that their puberty duration is longer.

## Introduction

Endocrine problems are common in children with Down syndrome (DS). These include mainly short stature, hypothyroidism, obesity, type 1 diabetes; low bone mineral density, and infertility (1). There are few studies regarding pubertal problems of children with Down syndrome. Studies on gonadal functions in men have shown progressive impairment in spermatogenesis and Sertoli cell functions (2). There are studies showing that menarche is generally similar to their healthy peers and their own mothers in girls with DS (3, 4). Our knowledge on pubertal problems in children with Down syndrome is based on studies conducted about three decades ago (5–7). As it is well known, puberty in children starts at a younger age as the trend of the century. Yet, little is known about the age of onset of puberty and the duration of puberty in children with Down syndrome. Puberty timing is affected by race, nutrition, genetic and environmental factors. Previously published longitudinal studies have shown a significant correlation between higher body fat and earlier pubertal development and menarcheal age, pubertal growth spurt, earlier development peak height velocity (PHV) in females (8). In a study, it was found that males with early sexual maturation were thinner and girls were fatter compared to their peers (9).

Our aim in this study was to determine whether the developmentof puberty in DS children was different from their healthy peers and from studies conducted two to three decades ago in DS children

## Material and methods

Our study was conducted by retrospective examination of the medical records of DS children (through screening ICD-10 codes). This is a cross sectional study. The file information of the patients was obtained retrospectively between January 2017 and December 2019. Nineteen in the study group were followed up from the clinic where Dr AG previously worked. All patients were examined at 3-4 months intervals.

Patients who applied to the clinic were examined after recording their complaints, birth weight, gestational week, and the presence of parental consanguinity. The height and weight were measured by stadiometer with bare feet and light clothing. Blood pressure measurement and systemic physical examination were performed in all patients. Stretched penile lengths (SPLs) and testicular volumes (TVs) were measured with Prader orchidometer in males. The stage of breast development in girls was evaluated by palpation. Pubic and axillary hair growth was examined in both genders, and the presence of clitoromegaly in girls was investigated. Pubertal staging was evaluated in both genders. Tanner stage I according to testicular volume: 1-3 ml; Stage II: 4-6 ml; Stage III: 8-10 ml; Stage IV: 15-20 ml; Stage V was accepted as >20 ml. The onset of secondary sexual characteristics as Tanner Stage II in a girl before the age of 8 years, or in a boy before the age of 9 years is accepted as precocious puberty (PP). If puberty started between the ages of 8-9 in girls and between the ages of 9-10 in boys, and progressed to Tanner III in less than six months, this condition was accepted as early-normal puberty (10). Age, height, weight and body mass index (BMI) standard deviations of the patients, were calculated using an online calculation tool called ceddcozum.com (11).

The findings were compared with the data of previous studies conducted on healthy children from our country (12, 13), and with previously published data of patients with DS (2, 3, 5–7, 14, 15).

Blood samples were taken for follicle stimulating hormone (FSH), luteinizing hormone (LH), total testosterone, estradiol (E2) levels only in patients with signs of precocious puberty. Bone age was determined using the Greulich and Pyle bone age atlas (16). Blood was drawn for dehydroepiandrosterone sulphate (DHEA-S) and 17-OH progesterone levels in patients with premature pubic or axillary hair growth. The presence of menarche was questioned in those with onset of puberty symptoms.

### Statistics

Analysis of data and statistical analysis; SPSS 22.0 (Statistical Package for the Social Sciences) program was used. In the evaluation of the data, descriptive statistical methods (mean and standard deviation for normally distributed data, median for non-normally distributed data, (interquartile range, IQR) (minimum and maximum value) were used. One-sample T test was used to compare the puberty stages and menarche ages of the patients in the study group with the ages of healthy patients and to compare with DS patients in the literature. A p value <0.05 was taken to indicate statistical significance.

### Ethics

Approval was obtained from the Zeynep Kamil Women and Children Hospital Ethics Committee (22.0.2020/160).

## Results

Of the 140 Down syndrome patients followed in our clinic, 51 pubertal children constituted the study group. Karyotype analyses revealed 47, XX + 21 in girls and 47, XY + 21 in boys. The mean age of 28 girls included in the study was 13.26 ± 2.3 years, and the mean age of 23 boys was 12.97 ± 2.5 years, and there was no statistical difference between them.

The follow-up period of the patients was 88.9 ± 42.8 (25–192) months in the whole group. Girls were followed for 82.8 ± 38 (31–156) months, while boys were followed for 97.7 ± 48 (25–192) months.

Among them, 13 girls (46%) and 5 boys (17%) were obese. There was no difference regarding the ages of onset of puberty, duration of puberty, and age at menarche between children with and without obesity. Malnutrition was not detected in any of the patients.

As expected, puberty started in girls (10.04 ± 0.8 years) earlier than boys (10.65 ± 1.22 years) (p=0.02) and was completed earlier (15.69 ± 1.22 years in girls, 16.33 ± 1.04 years in boys, respectively). It was observed that 9.3% of the children with DS completed their puberty. The ages of puberty onset and finish were compared with DS children in the literature and and the results were given in **Tables 1** and **2**.

**Table 1:** Comparison of ages at each pubertal stage and at the menarche of girls with Down Syndrome with their healthy peers and published girls with Down syndrome: One-sample T test results (n:28).

Tanner Stages	Girls with Down syndrome	Healthy peers in Ref. in 12	Healthy peers in Ref. in 13	Girls with Down syndrome in Ref. 5	Girls with Down syndrome in Ref. 3	Girls with Down syndrome in Ref. 6	Girls with Down syndrome in Ref. 7	Girls with Down syndrome in Ref. 16
	Age, years; p	Age, years; p	Age, years; p	Age, years; p	Age, years; p	Age, years; p	Age, years; p	Age, years; p
II	10.4±0.7	9.65; **p=0.03**	10.16; p=0.337	12.2; **p<0.001**				
III	12.8±1.9	10.10; p=0.07	11.72; p=0.348					
IV	13.7±1.0	11.75; p=0.142	12.97; p=0.474					
V	15.0±1.6	14.17; **p=0.008**	13.66; **p<0.001**	13.9; **p=0.008**				
Menarche	11.83±0.77	12.74; **p<0,001**	12.41; p=0.310		13.2**; p<0.001**	13.6; **p<0.001**	11.9; **p=0.032**	12.08; p=0.283

Statistically significant values are written in bold in the tables.

**Table 2:** Comparison of penile length and testicular volume of the patients according to the puberty stage with their healthy peers and published patients with Down syndrome: One sample T test results (n:23).

Tanner stages	Age, years	SPL of patients, cm	Testicle volume of patients, ml	Age, yr healthy peers (Ref 13)	Boys with Down syndrome in Ref. 14	Boys with Down syndrome in Ref. 5	Boys with Down syndrome in Ref. 2
II	12.1±2.3	7.7±1.4	4.8±0.9	11.76; p=0.648		13 years	SPL: 5.9±2.3 cm; **p=0.03.** TV:7.8±5.1 mL; **p<0.001**
III	13.2±1.5	8.5±0.7	90±1.4	12.8; p=0.503			SPL: 7.8±5.1cm; p=0.656 TV:11.0±4.8mL; p=0.295
IV	14.3±1.2	9.0±1.0	14.0±1.7	13.17; p=0.229			SPL: 8.8±1.3 cm; p=0.762 TV:13.0±4.4 mL; p=0.423
V	15.8±1.7	9.8±1.7	20.8±2.0	13.97; **p=0.046**	TV: 19.4±4.6; p=0.146	16.8 years SPL: 11.6 cm, p=0.054; TV: 11.8 mL, **p<0.001**	SPL: 8.0±2.1cm; **p=0.048.** TV:12.4±5.5mL; **p<0.001**

SPL, stretched penile length.

Statistically significant values are written in bold in the tables. TV, testicular volume.

Pubertal height gain of 16 children was calculated. It was determined that the girls gained an average height of 6.92 ± 4.8 cm and the boys 18.3 ± 7.2 cm. Mean height gain in girls after menarche was 1.15 ± 1.0 cm.

### Comparison with published studies in girls

Our results in girls were compared with the results of the study conducted with the age of pubertal onset in healthy children (**Table 1**). According to recently published study (12) in our country, breast development in DS girls started later than their healthy peers. Also, the age of Tanner stage V breast development was found statistically later than their healthy peers (12).

In our study, 18 girls with DS were found to have menarche. Age at menarche was found to be younger than the healthy peers (12). Comparing the age at menarche with previously published DS females, our patients were found to be younger than two former studies (3, 6), but there was no difference compared to other studies (7, 15) (**Table 1**). There was no difference regarding the age at menarche of obese (11.87 ± 0.7) and normal weight girls with DS (11.45 ± 0.5).

### Comparison with published studies in boys

The ages at pubertal onset and different pubertal stages in boys with DS were compared with the ages of the healthy group (13). It was found that boys with DS reached Tanner stage V testicular volumes at a later age than their healthy peers (**Table 2**).

Stretched penile length and TV of our patients were compared with those of published DS patients (2, 5, 14). Although, the SPL of our patients in stage II was longer from the patients in the literature, TVs of them were smaller (2). In addition, our patients in Stage V had larger TVs and longer SPLs (2, 5) (**Table 2**).

### Early normal puberty and true precocious puberty:

In two of our male patients, early normal puberty was found (at the ages of 9.07 and 9.3 years, respectively). Both patients were diagnosed with hypothyroidism and were euthyroid with L-thyroxin treatment. True precocious puberty was detected in three children (two males and one female) with DS (**Table 3**). All three patients had hypothyroidism. By the time of diagnosis with true precocious puberty, all three patients were on proper L-thyroxin treatment and were in the euthyroid state.

**Table 3:** Laboratory results and bone ages of patients with down syndrome and precocious puberty.

At the diagnosis of CPP	Case 1 (Female) 7.56 years old	Case2 (Male) 7.40 years old	Case3 (Male) 8.96 years old
LH, mIU/mL	0.16	0.38	0.61
FSH, mIU/mL	2.87	1.81	9.66
E2, pg/mL	23	–	–
TT, ng/mL	<0.13	0.13	0.1
Peak FSH, mIU/mL	37.03	8.61	36.29
Peak LH, mIU/mL	26.28	7.79	18.05
17OHP, ng/mL (N<2 ng/mL)	0.62	0.28	1.01
DHEAS, mcg/dL	60.8	96	8.96
Bone age, year	10.48	8.48	6.48

### Hypothyroidism:

It was determined that 36 (70%) of the DS children in puberty received L-thyroxin treatment. The mean age of onset of puberty in DS children with hypothyroidism was 10.3 ± 1.1 years and 16 ± 1.3 years at the end of puberty. There was no statistically significant difference between the age of pubertal onset and the age of completion of puberty in DS patients with and without hypothyroidism.

### Associated abnormalities

Congenital heart disease was detected in the neonatal period in 26 pubertal children with DS, and 18 of them had been operated. Adrenal insufficiency, epilepsy, type 1 diabetes, micropenis, and hypospadias were found in one patient each. One pubertal child had been operated for anal atresia.

A cardiac disease was found in 16 of the children with hypothyroidism. There were 12 children with hypothyroidism who were obese, but none of them had malnutrition.

## Discussion

In children with Down syndrome, endocrine disorders such as short stature, obesity, hypothyroidism, diabetes, and puberty problems are more common than their healthy peers (5, 17, 18).

The age of pubertal onset in general population has been decreased to approximately 0.3 years per 10 years in the last century. This was thought to be related to better nutrition and socioeconomic status of the children. In recent years, especially in girls living in America and Europe, breast development has been observed at an earlier age due to the increase in obesity and the effect of endocrine disruptors (19).

In our study, it was found that our female patients with DS entered puberty earlier than males, as expected. When compared with the results of Hsiang et al. (5), it was found that our patients entered puberty at an earlier age in both genders. This result may be related to the shift of the onset of puberty to younger ages.

In our study, it was found that the ages of boys with DS to reach Tanner stage II to IV were not different from their healthy peers, but they reached Tanner stage V testicular volumes at a statistically significant and later age compared to their healthy peers (13). The age of pubertal onset of our female patients with DS was similar with a study conducted with healthy girls in our country (13). However, compared to another recent study conducted with a higher number of healthy girls in our country (12), we found that breast development in DS girls started later and reached stage V in a longer duration than their healthy peers.

It has been found that DS boys generally enter puberty at ages similar to their healthy peers, but have higher gonadotropin levels, especially men with DS over 30 years of age (2, 3, 5, 20) This increase was found to be accompanied by a decrease in TV and softening of the testicles (20). In addition, progressive loss of function in Sertoli and Leydig cells, decrease in anti-müllerian hormone levels and increase in FSH have been shown in patients from infancy (21). These studies suggest that gonadal dysfunction starts after birth in men with DS and the deterioration increases with age. Penile length of our patients at the beginning of puberty and the last stage of puberty was found to be longer from patients with DS published in the literature. While the TVs of our patients in stage V were larger than those in two studies (2, 5), they were not different from another study (14). It has been found that the penis length of adult men with DS is shorter than healthy men (2).

Arnell et al. (3) showed that the average age of menarche is 13.2 years in 13 DS girls who reached puberty and was not different from their mothers. Gonadotropin and estradiol levels were within normal limits in girls with DS (3, 4). Goldstein et al. (6) showed that the mean age of menarche was 13.6 years in 15 DS women and found no differencefrom the control group. On the contrary, Takano et al. (7), found that the real age of menarche in DS girls was 11.9 ± 0.1 years, after adjusting the age of menarche for girls with DS for the age of non-menstruating girls, which was younger than the population average. Researchers thought that early menarche in girls with DS might be related to obesity. In our study, we did not find any difference regarding the age of menarche between obese and non-obese girls. However, the relation between obesity and earlier menarche seen in these patients needs to be further evaluated.

When the menarcheal age of our female patients with DS was compared with the findings of the literature, the age of menarche in girls with DS was found to be younger compared to studies with similar numbers of subjects (5, 6). However, no difference was found from the studies conducted with more subjects (7, 15). The four comparable studies are those that were published 2-3 decades ago. In addition, since we found that obesity contributed in half of our patients to a younger age at menarche, we think that menarche may be seen earlier as the trend of the century also in girls with DS (22).

Although no difference was found in boys in our study, it was found that the age to complete puberty for girls was higher than those in the literature (5).

True-PP (TPP) is very rare in patients with DS (3, 23). Pseudo-PP is related to hypothyroidism in patients with DS and early menarche with multi-cystic ovaries has been reported in girls with DS with hypothyroidism (24). All three of our patients with TPP were receiving treatment for hypothyroidism. However, all of them were in euthyroid state as the symptoms of puberty started. Pelvic ultrasonographic examination was normal in a female with TPP. Cranial magnetic resonance imaging (MRI) in one of the boys showed a hypoplastic pituitary gland (4.3 mm) for his age. In the other size, periventricular millimetric foci were detected in the supratentorial region, but the pituitary gland was normal (**Table 3**).

## Study limitations

Our study has some limitations. This is a single center, cross-sectional study with a relatively small number of patients. and its retrospective design is a limitation. Second, bone age, gonadotropin, and sex steroid measurements were not taken from our patients at the beginning of puberty and during puberty. However, the characteristics of puberty in patients with DS is a not well-studied topic, and we believe that our study makes a valuable contribution to the sparse literature with new insights into the secular trend of puberty in these patients.

## Conclusion

This study demonstrated that breast development starts later in girls with DS than their healthy peers, but girls with DS had menarche before their healthy peers and reach stage V breast stage later than healthy girls. While the age at pubertal onset in boys with DS is not different from healthy children, it was observed that the age of reaching the last stage of puberty is later than their healthy peers. However, we also emphasize that studies with larger numbers of patients are needed to better understand the puberty process in children with Down syndrome.

## Data availability statement

The raw data supporting the conclusions of this article will be made available by the authors, upon reasonable requested.

## Ethics statement

Written informed consent was obtained from the minor(s)’ legal guardian/next of kin for the publication of any potentially identifiable images or data included in this article.

## Author contributions

Concept and Design: AG. Data Collection or Processing: FE. Literature Search: AG and FE. Writing: AG and FE. All authors contributed to the article and approved the submitted version.

## Conflict of interest

The authors declare that the research was conducted in the absence of any commercial or financial relationships that could be construed as a potential conflict of interest.

## Publisher’s note

All claims expressed in this article are solely those of the authors and do not necessarily represent those of their affiliated organizations, or those of the publisher, the editors and the reviewers. Any product that may be evaluated in this article, or claim that may be made by its manufacturer, is not guaranteed or endorsed by the publisher.
